# Lactic Acid Fermentation of Arabinoxylan From *Nejayote* by *Streptococcus infantarius* ssp. *infantarius* 25124 Isolated From Pozol

**DOI:** 10.3389/fmicb.2018.03061

**Published:** 2018-12-18

**Authors:** Barbara Cooper-Bribiesca, Arturo Navarro-Ocaña, Gloria Díaz-Ruiz, Guillermo Aguilar-Osorio, Romina Rodríguez-Sanoja, Carmen Wacher

**Affiliations:** ^1^Departamento de Alimentos y Biotecnología, Facultad de Química, Universidad Nacional Autónoma de México, Mexico City, Mexico; ^2^Instituto de Investigaciones Biomédicas, Universidad Nacional Autónoma de México, Mexico City, Mexico

**Keywords:** *Streptococcus infantarius* ssp. *infantarius*, lactic acid bacteria, arabinoxylan, *pozol*, *nixtamal*

## Abstract

*Streptococcus infantarius* ssp. *infantarius* 25124 (*Sii-*25124) is a lactic acid bacterium (LAB) isolated from *pozol*, a refreshing beverage prepared by suspending fermented *nixtamal* (a thermal and alkali-treated maize dough) in water. Although *Lactobacillu*s are the predominant strains in fermented doughs, such as sourdoughs, and non-nixtamalized fermented maize foods, the *pozol* microbiota is markedly different. This may be the result of the nixtamalization process, which could act as a selective force of some strains. *Sii-*25124 has been reported as the main amylolytic LAB in pozol; starch is the primary carbon source on *nixtamal* since monosaccharides and disaccharides are lost during nixtamalization; however, non-amylolytic LAB counts are higher than amylolytic LAB in *pozol* after 24-h fermentation suggesting that another carbon source is being used by the former bacteria. Hemicellulose (arabinoxylan in maize) becomes available via nixtamalization and is subsequently metabolized by LAB. The aim of this work was to determine whether this bacterium is able to use arabinoxylan as the only carbon source in a defined medium containing arabinoxylan extracted from either *nejayote* (wash water produced during *nixtamal* preparation), or beechwood xylan. Xylanase activity in the presence of *nejayote* arabinoxylan (135.8 ± 48.7 IU/mg protein) was higher than that of beechwood (62.5 ± 19.8 IU/mg protein). Other enzymatic activities, such as arabinofuranosidase and acetyl esterase, were also detected, suggesting the adaptation of the bacterium studied to *nixtamal* dough. It was concluded that *Streptococcus infantarius* 25124 isolated from *pozol* was able to use arabinoxylans, which are present in nixtamal dough, so fermentation does not depend exclusively on free sugars and starch.

## Introduction

*Pozol* is one of the most important traditional pre-Columbian non-alcoholic beverages in southeastern Mexico, where it is consumed as part of the daily diet. It is made of fermented *nixtamal* (heat- and alkali-treated maize dough). To prepare *pozol*, maize is nixtamalized; to this end, white, yellow or *mestizo* corn is shelled and kernels are cooked with lime (0.6–1.2% dry maize weight) for 50–80 min, followed by a 12- to 14-h soaking in its own cooking liquor. Afterward, the product is washed with water two to three times and rubbed by hand to remove the pericarp and lime, and is then coarsely ground (Ulloa et al., 1987; Cañas et al., 1993). The resulting heterogeneous dough, also known as *masa* or nixtamalized *masa*, is shaped into balls, wrapped in banana leaves, and allowed to ferment for a period of time that varies from approximately 3 h up to 1 month or longer. A complex microbiota is found in the fermenting dough; the microbial ecology of *pozol* has been studied by Ampe et al. (1999), Ben Omar and Ampe (2000), Escalante et al. (2001), and Díaz-Ruiz et al. (2003). They found a variable diversity of species, which after 24 h of fermentation are reduced to strains belonging mainly to the genera *Streptococcus, Weissella, Enterococcus*, and *Lactococcus*. *Streptococcus infantarius* ssp. *infantarius* 25124 (*Sii-*25124) is the main amylolytic LAB (ALAB) of *pozol. Streptococcus infantarius* ssp. *infantarius* strains have also been isolated from traditional fermented dairy products for example maasai fermented milk and Fènè (fermented cow milk) (Jans et al., 2017). It has been reported as pathogenic and there is not yet a general method to discriminate pathogenic from non-pathogenic strains. Kaindi et al. (2018) isolated bacteria of the same subspecies from African dairy products, which were clearly different from the human strain *Streptococcus infantarius* ssp. *infantarius.* Coming from a fermented food, it is possible that our *Sii-*25124 is also different from human strains. *Streptococcus infantarius* ssp. *infantarius* (*Sii)* was previously classified as *Streptococcus bovis* biotype II/1, but it was differentiated through phenotypic and genotypic Schlegel et al. (2000, 2003) and sodA gene sequencing (Poyart et al., 2002; Romero et al., 2011). Members of the *Streptococcus bovis/Streptococcus equinus*, to which *Streptococcus infantarius* ssp. *infantarius* 25124 (*Sii-*25124) belongs, have been reported as pathogenic, associated mainly with colorectal cancer; however, it is also known that there are safe strains. The problem of detecting the pathogenic strains is presently a topic of arduous study (Papadimitriou et al., 2014; Jans and Bolieij, 2018). Compared to other ALAB, it shows a low amylolytic activity (130.7 U/g dry cell weight/h in MRS starch (De Man RogosaSharp medium), but a high specific growth rate (μ = 0.94/h); compared to *Lactobacillus fermentum* shows an amylolytic activity of 1,890 U/g dry cell weight/h in MRS starch and a specific growth rate of 0.35/h (Díaz-Ruiz et al., 2003).

During nixtamalization, a large proportion of soluble carbohydrates are removed. Sucrose concentration is reduced to 100–700 mg/100 g dry dough (Santillana, 1995), and maltose and glucose have each been detected at concentrations below 3.6 mg/100 g in dry dough (Ben Omar and Ampe, 2000); thus, starch is left as the main carbohydrate for microbial growth. However, maize pericarp contains hemicellulose and cellulose, both of which are structural carbohydrates, and *nixtamal* dough contains 6% total non-starch polysaccharides (NSP), by dry weight (Englyst and Hudson, 1996; Instituto Nacional de Nutrición Salvador Zubirán [INNSZ], 1996; Sanchez-Castillo et al., 1999).

*Nejayote* is the wastewater (wash water) resulting from nixtamalization. It is highly alkaline (pH 11–12) and contains more than 60% of alkali-soluble NSP from maize pericarp, which are mainly arabinoxylans. The information currently available on xylanase activity by LAB is scarce. Moreover, efforts to identify xylanolytic activity have detected that even in the presence of the *xyl* operon, the dairy starter culture strain *Lactococcus lactis* 210 displays a loss of function of the xylanase genes. In addition, while plant environmental isolates such as *L. lactis* NRRL B-4449 and *L. lactis* IO-1 retain the ability to metabolize xylose, neither of these two strains is able to grow in the presence of xylan or xylobiose (Erlandson et al., 2001). Only putative xylan-acting enzymes in *Streptococcus, Lactococcus, Lactobacillus*, and *Enterococcus*, and at least two in *Tetragenococcus* and one in *Leuconostoc*, have been found in databases (Uniprot, 2015); however, no functional xylanases from LAB have been reported.

Besides their importance as carbon and energy sources, xylan hydrolysis products may include xylooligosaccharides (XOs), which are considered to be prebiotics: wheat-bran XOs are utilized by *Bifidobacteria*, *Lactobacilli*, and *Pediococci* spp., as reported by Madhukumar and Muralikrishna (2012).

Because of the lack of monosaccharides and disaccharides in *nixtamal*, starch has been considered to be the main carbohydrate used by LAB during *pozol* fermentation. However, non-amylolytic LAB outgrow ALAB during *pozol* fermentation (Díaz-Ruiz et al., 2003), suggesting that another carbon source is being used by the former bacteria. During nixtamalization, the structural carbohydrates of maize, particularly hemicelluloses, should become more accessible for xylanolytic LAB due to their solubility in alkaline water.

There is little information regarding hemicellulose hydrolysis by LAB. The purpose of this work was to determine whether *Streptococcus infantarius* ssp. *infantarius* 25124 (*Sii-*25124) is able to use either maize arabinoxylan or beechwood xylan as carbon source, and whether it possesses the functional enzymes needed to hydrolyze both polysaccharides. We also aimed to characterize the fermentation of maize arabinoxylan extracted from *nejayote* by *Streptococcus infantarius* ssp. *infantarius 25124 (Sii-25124)*.

## Materials and Methods

### Microorganisms and Culture Conditions

*Streptococcus infantarius ssp. infantarius 25124 (Sii-25124)* isolated from *pozol* was recovered from cryo freezer vials containing glycerol, 50 μl in 5 mL APT broth (BD-DIFCO, United States) and incubated at 30°C for 24 h. A 10% (v/v) aliquot was then transferred to an 18-mL screw cap tube containing MRS broth prepared with 1% (w/v) beechwood xylan (Sigma-Aldrich, United States, 90% purity) instead of glucose (MRS-X). Samples were then incubated at 30°C for 18 h.

### Semiquantitative Xylanase Activity

The culture described above was used to streak MRS-Xylan (MRS-X) plates and incubated for 3 days at 28°C. Afterward, plates were flooded with Gram’s iodine and xylanase activity was determined semi-quantitatively based on the size of clear halos around colonies. *Cellulomonas flavigena* CDBB531 (Pérez-Avalos et al., 1996) was used as positive control.

### Arabinoxylan Extraction From *Nejayote* Liquor

Maize arabinoxylan was obtained from *nejayote* using a modification of the procedure described by Carvajal-Millan et al. (2007). *Nejayote* was sampled from a commercial mill located at the Niños Heroes market in Mexico City. Fifty kilograms of maize kernels were boiled in 1% (w/v) lime for 1 h, then left to stand for 12 h. Afterward, the liquid (*nejayote)* was drained out and maize kernels were processed to obtain the dough. *Nejayote* was recovered and filtered using a cloth, then centrifuged at 10,000 rpm and 20°C for 15 min to remove particles; the pH of the supernatant was adjusted to 5 with 3N HCl, and absolute ethanol was added with gentle stirring to a final concentration of 65% (v/v). Afterward, samples were incubated overnight at 4°C. The precipitated polymer was filtered and dried by adding 0.5 volumes of ethanol and 0.25 volumes of acetone per volume of filtrated *nejayote*. The dry residue was then dissolved in a minimum volume of hot distilled water and freeze-dried. Fourier Transform Infrared Spectroscopy (FIT-IR) was performed by the Analytical Services Unit at Faculty of Chemistry, UNAM, on dry samples of the arabinoxylan extracted, to confirm the nature of this chemical.

### Growth Kinetics on Beechwood Xylan and *Nejayote* Arabinoxylan

One-hundred-milliliter flasks containing 45 mL of HSH-defined medium broth (Owens and Keddie, 1969; Nuraida et al., 1992; Westby et al., 1993; Zaunmüller et al., 2006) were prepared with either 1% (w/v) beechwood xylan (Sigma-Aldrich, United States) or 1% (w/v) *nejayote* arabinoxylan, each inoculated with 5 mL of the prepared inoculum and incubated without stirring at 28°C. Aliquots were collected at 3-h intervals from baseline to 12 h, then at 24 h at 48 h, to evaluate growth. In each time point, aliquots were centrifuged (10,000 rpm, 5 min) and the supernatant was used to measure sugar concentration and enzymatic activities. Each sample was collected after stirring, since *nejayote* arabinoxylan is not completely solubilized.

### Growth Evaluation

Growth of the strain studied was evaluated by plate count on MRS agar (BD-DIFCO, United States), incubating for 24 h at 30°C, and the results were reported as log CFU/mL. Data were obtained from biological triplicates. The specific growth rate was determined from a regression analysis of the experimental data with the Logistic and Gompertz models (Zwietering et al., 1990; Baty and Delignette-Muller, 2004).

### Total and Reducing Carbohydrates

Reducing sugars were analyzed by the dinitrosalicylic acid (DNS) method (Miller, 1959) using a xylose standard curve (0.025–0.6 mg/mL, Sigma-Aldrich, United States). Properly diluted supernatants were tested for total sugar content by the phenol sulfuric acid method as described by Dubois et al. (1956) and expressed as xylose equivalents; sugar content was calculated using a calibration curve spanning from 0.01 mg/mL to 0.1 mg/mL xylose.

### Fermentation Metabolites

The HPLC parameters were as follows: Waters AF equipment, Waters binary pump 1525, Waters Autosampler 2707, Waters dual Absorbance detector 2487, Empower software, and Aminex-HPX-87H column (300 mm × 7.8 mm). The mobile phase was 0.05N H_2_SO_4_. The flow rate was 0.6 mL/min with a 30-μL injection volume and a working temperature of 30°C. A UV detector was used for the fermentation products (lactic and acetic acids, ethanol). Each compound was identified by comparing its retention time vs. standards (Sigma-Aldrich, United States). Concentrations were calculated by comparing peak areas against calibration curves prepared separately as follows: ethanol, 0.2–1% (v/v); lactic acid, 0.2–1.2 mg/mL; acetic acid, 0.012–0.0042 mg/mL.

### Xylanase Activity

#### Overall Xylanase Activity

Xylanase activity was measured according to Rickard and Laughlin (1980) using 1% (w/v) beechwood xylan as substrate. A xylanolitic bacterium, *Cellulomonas flavigena* CDBB531 (Pérez-Avalos et al., 1996), was used as positive control. Activity was determined by measuring the increase in reducing sugars released during a 20-min incubation at 40°C in a solution containing 500 μl supernatant, 500 μl citrate–phosphate buffer (pH 6.8) and 500 μl beechwood xylan (1% w/v) in distilled water (Bailey et al., 1992). The reaction was stopped by adding 1.5 mL of DNS, then shaking and boiling the samples for 5 min. Reducing sugars were measured by spectrophotometry (Spectronic 21D, Milton Roy, United States) at 540 nm using a xylose standard (Sigma-Aldrich, United States) (Miller, 1959) and a calibration curve ranging from 0.025 mg/mL to 0.6 mg/mL. Protein concentration was determined according to the method of Bradford (1976) using bovine serum albumin as reference standard. One international unit (IU) of activity was defined as 1 μmol of xylose or xylose equivalents released per minute under the assay conditions described. All tests were carried out in triplicate, and average values were recorded.

#### β-Xylosidase Activity

The activity of β-xylosidase was determined by incubating 25 μL of the fermentation supernatant with 100 μL of 1 mg/mL p-nitrophenyl-D-xylopyranoside (PNP-xyl, Sigma-Aldrich, United States) in citrate-phosphate buffer (0.1 M and 0.2 M, respectively) (pH 6.8) at 40°C for 20 min. The reaction was stopped by adding 75 μL of 0.25 M Na_2_CO_3_, and the *p*-nitrophenol released was measured using a spectrophotometer (Spectronic 21D, Milton Roy, United States) at 405 nm, reagent and substrate blanks were employed. One unit of β-D-xylosidase activity was defined as the amount of enzyme that released 1 μmol of *p*-nitrophenol per milliliter under the assay conditions.

#### Supplementary Xylanase Enzymes

Additional enzymatic activities were assayed with the appropriate PNP substrates, all of which were sourced from Sigma-Aldrich, United States (pNP-β-D-glucopyranoside; pNP-α-D-glucopyranoside; pNP-β-arabinofuranoside, pNP-α-arabi nofuranoside; pNP-β-D-galactopyranoside, pNP-α-D-galacto pyranoside, pNP-α-xylopyranoside, and pNP-4-nitrophenyl-acetate), using the same conditions as described for β-xylosidase activity.

#### Statistical Analysis

The differences between the xylan and arabinoxylan fermentations were assessed through the differences between the areas under the curve and comparing the differences between the average values with a Student’s *t*-distribution (α = 0.01).

## Results

### Nature of the Chemical Extracted From *Nejayote*

**FIGURE 1:**
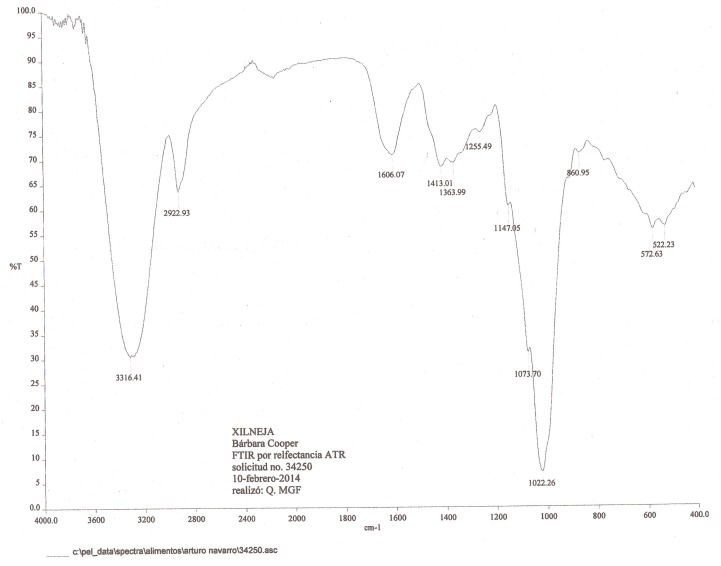
FIT-IR spectra of the chemicals extracted from *nejayote*.

Arabinoxylan from *nixtamal* was used as the substrate to assess whether *Sii-*25124 was able to use it as the only carbon source. It is soluble in alkali, so it is found in the dough and also at high concentrations in *nejayote*, i.e., the nixtamalization wash water. The FIT-IR spectrum of the chemical extracted from *nejayote* showed the typical signals of hemicelluloses: a strong signal at 3316.41/cm from -OH stretching; at 1147/cm from C-O and C-O-C stretching and C-OH bending of arabinoxylan-hemicelluloses; at 1022/cm from C-OH bending of hemicelluloses; and at 860/cm from β-glucoside bonds in hemicellulose and a furanoid ring (arabinofuranoside) (Figure 1). The typical signals of absorbed water, protein or lignin were not detected at the 1700-1500 and 1654-1539 regions. The spectrum resembled the one obtained by Oliveira et al. (2010) for corncob arabinoxylan. This product showed only one signal on HPLC and was assayed for reducing sugars with only 7.71 μg per mg arabinoxylan; therefore, it contains no other free sugars and can be used to assess its consumption.

### Growth of *Sii-*25124 on Beechwood Xylan and *Nejayote* Arabinoxylan in HSH Broth

**FIGURE 2:**
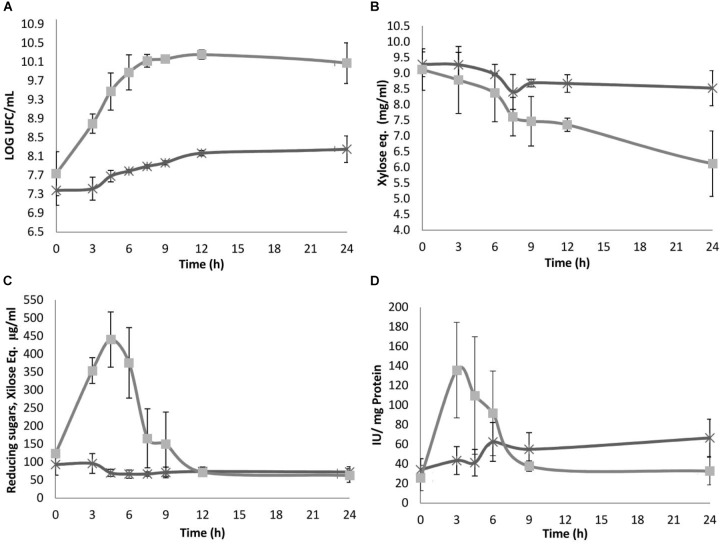
**(A)** Growth, **(B)** total carbohydrates, **(C)** reducing sugars, and **(D)** specific xylanase activity; concentration during fermentation by *Streptococcus infantariu*s ssp. *infantarius* 25124 in HSH broth spiked with 1% beech wood xylan (X) and *nejayote* arabinoxylan (□) as carbon sources. Results are means of three separate fermentations; standard deviations are shown as vertical lines. There were statistically significant differences between the treatments (*nejayote* arabinoxylan vs. beechwood xylan) (α = 0.01).

*Sii-*25124 was able to grow in both xylan types. Significant differences were observed in growth; reducing and total sugar consumption rates; and enzymatic activity induced by xylan and arabinoxylan. No lag phase was observed in arabinoxylan; in contrast, for beechwood xylan the log phase started after 3 h (Figure 2A). The logistic model resulted in the better goodness of fit than the Gompertz model for both microorganisms. The criterion to differentiate between both models was the sum of squared residuals, expressed as SSR = $\sum_{i\mathrm{=1}}^{n}$(ypred - yexp)${}_{i}^{2}$, where *y pred* and *y exp* are the predicted and experimental values of microbial growth, respectively. Thus, the values of μ max were 0.109 h^-1^ (SSR = 3.183 ⋅ 10^-3^, *r*^2^ = 0.9683) for arabinoxylan, and 0.0267 h^-1^ (SSR = 9.548 ⋅ 10^-4^, *r*^2^ = 0.9551) for beechwood xylan.

On HSH beechwood xylan, *Sii-*25124 population increased one log unit after 48 h. *Sii-*25124 grown in *nejayote* arabinoxylan displayed a growth rate four times as high as in beechwood xylan and reached the stationary phase after 12 h (with a population increase of nearly 3 log units in 12 h). After 24 h, the culture containing *nejayote* arabinoxylan entered the death phase concurrent with a decrease in the population vs. cultures grown in beechwood xylan, which showed a continuous linear growth (Figure 2A). The strain was able to grow in HSH-glucose and HSH-xylan, but not in HSH medium with no carbon source (data not shown), hence demonstrating that growth on arabinoxylan results from the use of this carbon source. The pH of both cultures remained constant at 6.8, likely because the HSH medium contained phosphate buffer pH 6.8.

### Total Carbohydrates

Samples grown in *nejayote* arabinoxylan displayed a rapid decrease in total carbohydrate concentration, from 9.11 to 5.10 mg/mL (56% consumption), after 24 h. Samples grown in beechwood xylan showed a decrease in total carbohydrate concentration (from 9.28 to 7.87 mg/mL; 15%) after 24 h (Figure 2B). These results suggest a lower consumption of beechwood xylan vs. *nejayote* arabinoxylan, reflected as a lower growth rate in the culture medium (Figure 2A).

### Reducing Carbohydrates

Reducing carbohydrates were measured to account for the production and consumption of sugars, a feature coupled to enzymatic activity. In samples grown in beechwood xylan, the reducing sugar concentration after 3 h ranged from 93 to 61.9 μg/mL, with no reducing sugar accumulation during the remaining fermentation time (Figure 2D), when a constant concentration of 60 μg/mL was reached. A higher hydrolysis rate was observed in samples grown in *nejayote* arabinoxylan fermentation in which the carbohydrate concentration increased 3.6-fold, from 123.3 (initial) to 440.2 μg/mL, after 4.5 h of fermentation, indicating a much higher enzymatic activity related to hydrolysis and the production of reducing sugars (Figure 2D). These sugars were consumed until they reached a minimum average concentration of 58.1 μg/mL after 12 h of culture.

### Fermentation Products

**FIGURE 3:**
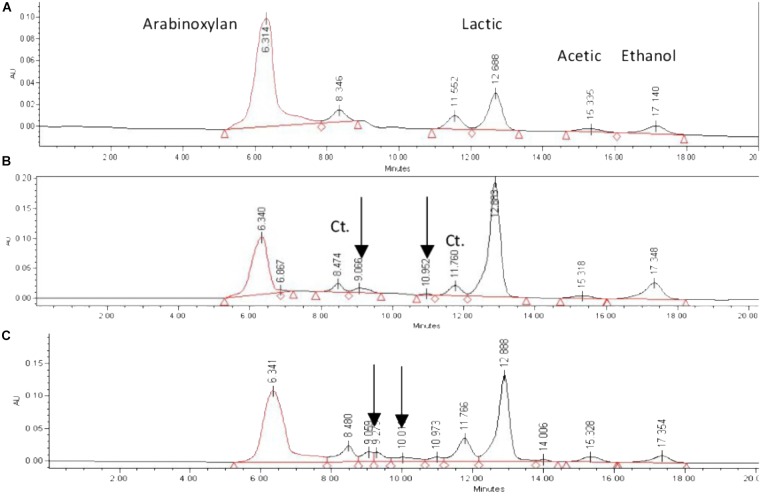
HPLC profiles of fermentation by *Streptococcus infantariu*s ssp. *infantarius* 25124 in HSH broth spiked with *nejayote* arabinoxylan as carbon source. Fermentation times: **(A)** 3 h, **(B)** 12 h, **(C)** 30 h. Retention times: arabinoxylan 6.3 min, lactic acid 12.8 min, acetic acid 15.3 min, ethanol 17.3 min. Unknown metabolites shown with arrows. Constant-area peaks indicated with Ct.

Fermentation products were evaluated by HPLC, with two main chemicals detected: lactic acid and ethanol; acetic acid was detected in negligible concentrations (Figure 3).

**FIGURE 4:**
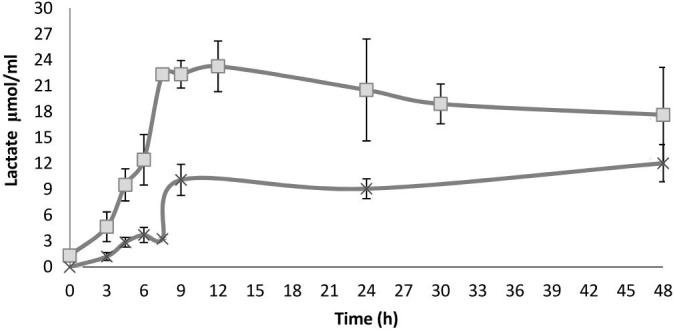
Lactate concentration along fermentation in HSH broth spiked with 1% beech wood xylan (X) and *nejayote* arabinoxylan (□) as the carbon sources and inoculated with *S. infantarius* ssp. *infantarius* 25124. Results are means of three independent separate fermentations; standard deviations are shown as vertical lines. There are statistically significant differences between the treatments (*nejayote* arabinoxylan vs. beechwood xylan).

The analysis of fermentation supernatants by HPLC revealed that the area of two peaks (retention time 8.4 and 11.6 min, respectively) remained constant from the beginning of the fermentation. Two additional peaks were detected after 4.5 h of fermentation, not matching any of the monosaccharide standards, and remained until the end of the fermentation (indicated with arrows in Figure 3). The overall trend observed in fermentation products was that metabolite concentrations increased and total carbohydrate concentration decreased as the culture proceeded (Figures 2B, 4).

Based on the fermentation products detected, *Sii-*25124 metabolizes sugars through heterolactic fermentation via a 6-phosphogluconate (6PG) pathway. This metabolic pathway has been observed in LAB: *Enterococcus mundtii* QU 25, *Lactococcus lactis* IO-1, and *Lactococcus lactis* ssp. *lactis* IL1403) (Bolotin et al., 2001; Tanaka et al., 2002; Abdel-Rahman et al., 2011a,b). Lactic acid and ethanol were produced in the early stages of fermentation, reaching peak concentrations after 7.5 h in *nejayote* arabinoxylan cultures (24.1 ± 0.23 μmol/mL ethanol; 23.28 ± 2.93 μmol/mL lactic acid), and after 9 h in beechwood xylan cultures (6.0 ± 0.75 μmol/mL ethanol; 10.1 ± 1.22 μmol/mL lactic acid), remaining constant until 12 h in both cases (Figure 4). Ethanol concentration did not increase. Based on the calculation of the theoretical phosphogluconate pathway, one mol of lactate plus one mol of ethanol or acetate were expected per mol of pentose fermented. The ratio remained unchanged up to 12 h. Although homolactic LAB such as streptococci and pediococci use the hexose monophosphate pathway, the 6PG path is followed with pentoses (Kandler, 1983). Additionally, there was a close correlation between reducing carbohydrate consumption and lactic acid production, which further confirmed that the sugars released were being fermented (Figures 2C, 4).

### Evolution of Extracellular Enzymatic Activities

#### Xylanase Activity

On *nejayote* arabinoxylan the activity increased almost twofold, with a peak of 135.8 ± 48.7 IU/mg protein after 3 h of fermentation (Figure 2C). Thereafter, the activity decreased gradually and remained constant between 9 and 24 h. The magnitude of xylanase activity was consistent with the amount of reducing sugar produced during fermentation. As the substrate decreased, so did the enzymatic activity and the resulting reducing sugars, reaching a minimum concentration at the end of the fermentation phase (Figure 2D).

However, on beechwood xylan, the peak activity was detected after 6 h of fermentation, when the activity reached 62.5 ± 19.8 IU/mg protein; it decreased by only 26% at the end of the fermentation (Figure 2C).

#### β-Xylosidase and Supplementary Xylanase Enzymes

Supplementary enzymes and β-xylosidase activity were also assayed. The activity of each of these enzymes was induced separately in the presence of each xylan type, with a more rapid induction found in samples containing *nejayote* arabinoxylan, except for β-xylosidase, whose activity was higher in beechwood xylan. This may be explained by the non-branched structure of this arabinoxylan, making growth dependent on the depolymerization of linear xylan. The highest specific activities were detected for β-arabinosidase, β-galactosidase, β-glucosidase, and acetyl xylan esterase (Table 1), in the presence of *nejayote* arabinoxylan, in which the attached acetyl groups must first be cleaved before endoxylanase can act upon the linear structure from which xylo-oligomers are produced.

**Table 1:** Maximum activities of xylanase supplementary enzymes detected on HSH with *nejayote* arabinoxylan and beechwood xylan inoculated with *Sii-*25124 and incubated at 30°C.

	*Nejayote* arabinoxylan	Beechwood xylan
**α-Xylosidase^a^**	8.29 IU/mg prot (24 h)	5.71 IU/mg prot (24 h)
**β-Xylosidase^a^**	3.17 IU/mg prot (4.5 h)	4.43 IU/mg prot (0 h)
**α-Arabinosidase^a^**	9.08 IU/mg prot (24 h)	5.15 IU/mg prot (9 h)
**β-Arabinosidase^b^**	3.99 IU/mg prot (0 h)	5.26 IU/mg prot (6 h)
**α-Glucosidase^c^**	6.65 IU/mg prot (24 h)	5.20 IU/mg prot (9 h)
**β-Glucosidase^a^**	8.91 IU/mg prot (24 h)	5.21 IU/mg prot (9 h)
**Acetyl-esterase^a^**	33.4 IU/mg prot (4.5 h)	9.13 IU/mg prot (24 h)
**α-Galactosidase^a^**	5.35 IU/mg prot (0 h)	4.46 IU/mg prot (4.5 h)
**β-Galactosidase^a^**	6.72 IU/mg prot (6 h)	4.18 IU/mg prot (0 h)


*Incubation times in parenthesis. IU, μmol of liberated PNP/min^∗^mL. ^*a*^There were statistical differences along fermentation time between treatments (*nejayote* arabinoxylan vs. beechwood xylan). ^*b*^There was statistical difference between treatments on 3, 6, and 24 h. ^*c*^There was statistical difference between treatments except on 9th hour (α = 0.01).*

High xylanolytic, β-galactosidase, acetyl-esterase, and β-xylosidase activities were observed during the initial hours (between 0 and 6 h) of the fermentation process in the presence of *nejayote* arabinoxylan; however, the activity of other supplementary enzymes increased by the end of the fermentation (Table 1). The enzymatic behavior in samples grown in beechwood xylan differed, as some of the supplementary enzymes reached a peak activity at 9 h of fermentation, when the peak lactate concentration was observed (Figure 4). Alfa-xylosidase and acetyl-esterase activities peaked at 24 h, toward the end of the fermentation.

## Discussion

The outer layers of maize kernels must be pretreated to make them accessible to cellulolytic and xylanolytic enzymes during fermentation. This pretreatment step is a bottleneck in their consumption by microorganisms and in the bio-processing of lignocellulosic materials for the production of biofuel, prebiotics or any other biological products (Alonso et al., 2001). To address this biological barrier and foster fermentation, either enzymatic hydrolysis by cellulases and hemicellulases (xylanases and supplementary enzymes) or chemical or physical hydrolysis is required.

An example of this process is maize nixtamalization. This has been used by Mesoamerican indigenous populations since Pre-Columbian times. This heat-alkali pretreatment causes discontinuity in the lignin structure by cleaving the bonds between lignin and carbohydrates, and removing acetyl and other acid groups, hence making them accessible to enzymes (Agbor et al., 2011). Overall, arabinoxylan is the second largest fermentable source of carbohydrates, only after starch (71.7% starch and 6.2% hemicellulose in the whole maize kernel (Watson and Ramstad, 1987). The thermal treatment also solubilizes arabinoxylan; therefore, this chemical is found in the resulting *nixtamal* dough, hence increasing the availability of carbon sources for microbial growth, as well as in the wash water (*nejayote*). Because of this enhanced bioavailability, the consumption of arabinoxylan in the dough by xylanolytic LAB strains may proceed via arabinoxylan hydrolysis, a process releasing sugars that become available for fermentation.

To prove this hypothesis, arabinoxylan was extracted from *nejayote* and used as the sole carbon source in HSH-defined medium. Growth, total and reducing carbohydrate content, and lactic and ethanol production were measured; *Streptococcus infantarius* ssp. *infantarius* 25124 (*Sii-*25124) underwent vigorous growth in the presence of arabinoxylan. This strain, which predominates during pozol fermentation, is homo-fermentative, but follows the 6PG pathway when it consumes pentoses. This work revealed that this microorganism was able to grow by hydrolyzing arabinoxylan.

Our results demonstrate that *Sii-*25124 was able to grow in the presence of arabinoxylan, inducing enzymatic activities that are needed for extracellular hydrolysis to facilitate fermentation. The high acetyl-esterase activity observed at the beginning of *nejayote* fermentation shows the adaptation of this strain to nixtamal dough arabinoxylans, as growth in a different xylan source, i.e., beech wood xylan, resulted in less efficient hydrolysis and carbohydrate fermentation. The high standard deviation values obtained for *nejayote* arabinoxylan are attributed to the heterogeneous nature of this substrate.

Xylanase activity was directly related to the growth of *Sii-*25124. As its activity in the presence of *nejayote* arabinoxylan became constant (from 9 h onward), the culture entered the death phase. This result contrasts with xylanase activity in beechwood xylan, which remained constant up to 24 h.

However, catabolic repression may inhibit *Sii-*25124 xylanase. In samples grown in beechwood xylan, total sugar consumption was slow, allowing the culture to continue growing and xylanase activity to remain constant throughout fermentation, with minimum lactate accumulation; when grown in *nejayote* arabinoxylan, it showed high xylanase activity that produced reducing carbohydrates and supplementary enzymes. Reducing sugars were quickly depleted, lactate and ethanol concentrations increased, and then xylanase activity decreased to a minimum value, while some of the supplementary enzymes reached a peak activity at 24 h (Table 1). This would allow *Sii-*25124 to utilize the different sugars produced and grow; the culture, however, started to decline.

This finding suggests that the differences observed in growth and enzyme production with xylan and arabinoxylan arise from their structural characteristics, but also point to that *Sii*-25124 possesses the ability to adapt to an hemicellulolytic environment. Hence, the prevalence of *pozol* fermentation by this strain may be based on its adaptive ability to utilize several complex carbon sources.

Lactic acid bacterium have been found in bioconversion reactions involving the transformation of hemicellulose fractions to lactic acid, mainly in genetically modified strains. Hu et al. (2011) achieved the direct conversion of xylan to lactic acid using a *Lactobacillus brevis* strain transformed with a xylanase gene. To allow a one-step hydrolysis and fermentation of lignocellulosic substrates, Moraïs et al. (2013) employed a *Lb. plantarum* strain engineered with genes coding for a GH6 cellulase and a GH11 xylanase to produce the respective enzymes, which worked synergistically. Xylanases are also involved in the production of XOs and arabinooligosaccharides (AXOs) that, according to Grootaert et al. (2007), have potential beneficial effects in humans as prebiotics and soluble dietary fiber (Azevedo-Carvalho et al., 2013). It is commonly accepted that diet has a major influence on the gut microbial community, Rivière et al. (2016) and Li et al. (2017) reported that consuming foods that contain prebiotics such as inulin-type fructans (ITF) xylan and arabinoxylan oligosaccharides (AXOs), favored the growth of Bacteroides and of bifidobacteria in colon, contributing to human physiological functions and health. *Bifidobacterium* spp., one the most important probiotic groups of gut bacteria, grow in the presence of XOs and AXOs as carbon sources that facilitate the prebiotic functions of these bacteria (Wang et al., 2010; Amaretti et al., 2013) which include production of antioxidants, vitamin B, conjugated linolenic acids, protection against pathogens and stimulation of the immune system; AX and AXOs have also shown to cause a butyrogenic effect (Rivière et al., 2016).

Some *Sii* strains have been associated with traditional African dairy and plant-based fermented foods, so these should be compared with the *Sii-*25124 strain isolated from *pozol*. *Sii-*25124 is capable of growing in the presence of starch and sucrose, among other carbohydrates, as concluded from API 50CH (Díaz-Ruiz et al., 2003). It is important to stress that although *Lactobacillu*s are the predominant strains in fermented doughs, such as sourdoughs, and non-nixtamalized fermented maize foods (Corsetti and Settani, 2007; De Vuyst et al., 2009; Iacumin et al., 2009), the *pozol* microbiota is markedly different. This may be the result of the nixtamalization process, which could act as a selective force of some strains.

## Conclusion

We have shown that *Streptococcus infantarius* ssp. *infantarius* 25124 (*Sii-*25124) grows in cultures added with arabinoxylan from *nejayote*, besides being the most ALAB in *pozol*, displaying its ability to prevail in the fermentation of this dough. Xylanase and supplementary enzymes were produced when exposed to xylan; β-arabinosidase, β-galactosidase, β-glucosidase, and acetyl xylan esterase were the most active enzymes. These results show that besides starch, xylan and arabinoxylan serve as substrates for lactic acid bacteria fermentation of *pozol* dough.

## Author Contributions

CW is the tutor of the student, she supervised the experimental procedures and the writing of the manuscript. BC-B was responsible for the experimental procedures and the writing of the article. AN-O, RR-S, GD-R, and GA-O undertook some of the experiments and reviewed the manuscript.

## Conflict of Interest Statement

The authors declare that the research was conducted in the absence of any commercial or financial relationships that could be construed as a potential conflict of interest.
